# Decentralized pandemic response and health equity: an analysis of socioeconomic disparities in COVID-19 mortality in Japan

**DOI:** 10.4178/epih.e2025049

**Published:** 2025-08-28

**Authors:** Hasan Jamil, Aminu Abubakar Kende, Shuhei Nomura, Fumiya Inoue, Takao Suzuki, Stuart Gilmour

**Affiliations:** 1 Graduate School of Public Health, St. Luke’s International University, Tokyo, Japan; 2 Division of Population Data Science, National Cancer Center Institute for Cancer Control, Tokyo, Japan; 3 Keio University Global Research Institute (KGRI), Tokyo, Japan; 4 Department of Health Policy and Management, Keio University School of Medicine, Tokyo, Japan

**Keywords:** COVID-19, Mortality, Socioeconomic factors, Healthcare disparities, Japan

## Abstract

**OBJECTIVES:**

Global data often link greater socioeconomic deprivation to higher coronavirus disease 2019 (COVID-19) mortality. However, whether decentralized governance can mitigate this disparity by enabling tailored, equitable local responses remains unclear. We assessed whether Japan’s decentralized pandemic response moderated the association between area-level socioeconomic deprivation and COVID-19 mortality across municipalities.

**METHODS:**

We analyzed 20,760 COVID-19 deaths from all Japanese municipalities during 2020-2021. We computed standardized mortality ratios using national age-specific and sex-specific rates to derive expected counts. We then fit a Bayesian spatial Poisson regression model with the log of expected counts as an offset to estimate smoothed relative risks (RRs). The Area Deprivation Index (ADI) represented the primary predictor; structured and unstructured random effects captured spatial correlation and residual variability.

**RESULTS:**

Mapping of smoothed RRs, categorized into quintiles, revealed higher mortality risk in northern, central, and western municipalities, with lower risk in southern and scattered central regions. Contradicting global trends, deprivation and COVID-19 mortality demonstrated an inverse association (ADI coefficient, -0.095; 95% credible interval, -0.173 to -0.018), indicating that more deprived municipalities exhibited lower RRs for COVID-19 mortality (9.1% reduction per 1-unit increase in ADI).

**CONCLUSIONS:**

The inverse relationship between area deprivation and COVID-19 mortality in Japan contrasts with global patterns. Although Japan’s decentralized health system ensured equitable access to COVID-19 treatment, lower mortality in more deprived areas likely reflects additional protective factors, including population density patterns and community-specific adaptations. These findings underscore the complex interplay between socioeconomic conditions and health outcomes during global health emergencies.

## GRAPHICAL ABSTRACT

[Fig f6-epih-47-e2025049]

## Key Message

Japan exhibited an inverse COVID-19 mortality pattern: 9% lower risk per unit increase in area deprivation, contrasting with patterns elsewhere. This occurred where universal healthcare access and decentralized public health infrastructure operated in a geographic context of rural, lower-density deprived areas. The convergence of health system characteristics, population distribution, and social factors likely produced this divergence from typical pandemic inequities.

## INTRODUCTION

The coronavirus disease 2019 (COVID-19) pandemic has profoundly impacted global health [1,2], resulting in more than 700 million cases and 7 million deaths worldwide [2]. The pandemic has exposed and exacerbated existing health disparities, with evidence suggesting that members of marginalized groups and those of lower socioeconomic status are disproportionately affected [3]. It is well documented that individuals living in areas of higher deprivation often experience worse health outcomes, including higher mortality rates [4]. This pattern has also been observed for COVID-19, with more deprived areas showing higher rates of infection and mortality, although this finding has not been universal [5].

By September 2022, Japan had confirmed approximately 21 million cases (about 17% of the population) and roughly 44,000 deaths. The country adopted a decentralized approach to its pandemic response. Local entities, particularly public health centers, played a central role in implementing essential public health interventions, including conducting testing, tracing infections, enforcing isolation, managing patient care, and disseminating information to the public [6]. Although this strategy posed challenges for maintaining national uniformity and coordinating pandemic measures, it enabled rapid, locally tailored responses aligned with regional needs and conditions [7,8]. Japan’s approach appears to have been highly effective: despite its hyper-aging society, COVID-19 mortality was approximately 57.7 deaths per 100,000 population—markedly lower than in other high-income countries such as the United States (341.11 per 100,000), France (254.68 per 100,000), and Germany (203.16 per 100,000). This striking difference makes it crucial to investigate whether Japan’s decentralized strategy helped mitigate the strong association between socioeconomic deprivation and COVID-19 mortality observed elsewhere [9].

In this study, we examine how Japan’s decentralized pandemic response may have influenced the relationship between regional socioeconomic differences and COVID-19 mortality rates by applying a spatial regression model to municipal-level data on socioeconomic deprivation and COVID-19 mortality.

## MATERIALS AND METHODS

This study examined municipality-level COVID-19 mortality across Japan in 2020 and 2021. Mortality data were obtained from the Ministry of Health, Labour and Welfare’s Vital Statistics of Japan, based on place of residence and identified using International Classification of Diseases code U07.1; data were stratified by sex and 5-year age group. We chose this timeframe to avoid confounding from the vaccination program that began at the end of 2021. Population data for 2020, also stratified by age and sex, were obtained from the System of Social and Demographic Statistics and used to standardize mortality rates [10].

### Standardized mortality ratio

We calculated the standardized mortality ratio (SMR) for each municipality. Indirect standardization was used because many municipalities had age-sex strata with zero deaths. Expected deaths were computed by multiplying the national age-specific and sex-specific COVID-19 mortality rates for the entire Japanese population in 2020-2021 by the corresponding population counts in each municipality. SMRs were then calculated as the ratio of observed to expected deaths, enabling comparisons adjusted for age and sex.

### Area Deprivation Index

Our primary explanatory variable, the Area Deprivation Index (ADI), measured socioeconomic deprivation at the municipality level. The ADI, originally developed by Nakaya et al. [11,12], integrates 8 aggregated municipality-level indicators: the proportions of (1) elderly couple households, (2) elderly single‑person households, (3) single‑mother households, (4) rented dwellings, (5) unemployed persons, (6) sales and service workers, (7) agricultural workers, and (8) blue‑collar workers. Each municipality received an ADI score on a scale of 0 to 10, with higher scores indicating greater deprivation. We obtained the precomputed municipality-level ADI dataset via direct data transfer from the Tomoki Nakaya Lab to the Division of Population Data Science, National Cancer Center Japan Institute for Cancer Control.

### Spatial autocorrelation and cluster detection

We first quantified global spatial autocorrelation in municipal SMRs using the Moran’s *I*, with significance evaluated via 9,999 Monte Carlo permutations. We then computed local indicators of spatial association (LISA) to identify municipalities forming clusters. LISA significance was set at a two-sided alpha of 0.05.

### Statistical analysis

We used a Bayesian spatial Poisson regression model to estimate the association between COVID-19 mortality and socioeconomic deprivation. Observed deaths were modeled as a Poisson outcome with the logarithm of expected deaths as an offset, and relative risk (RR) was estimated as a function of ADI. Spatial correlation between municipalities was addressed using both structured and unstructured random effects. Structured spatial effects were modeled with the Besag specification, accounting for relationships among neighboring municipalities. Unstructured random effects captured non-spatial, area-specific variability. Gaussian priors with large variances were assigned to fixed effects, and log-gamma priors were used for the precision parameters of random effects. Spatial relationships were constructed in 2 stages. First, a Queen contiguity matrix was created, linking municipalities that share at least 1 boundary point. Second, each geographically isolated municipality (principally islands) that lacked land neighbors was connected to its single nearest municipality, identified by the shortest centroid-to-centroid distance; this link was made reciprocal. The resulting augmented adjacency matrix was then used in the Besag spatial random-effects model. To visualize risk, we drew 6,000 samples from the posterior distribution of the log-linear coefficient for the ADI. For each selected ADI score, every coefficient sample was multiplied by that score and exponentiated to convert the log effect into a RR. This calculation was repeated for 100 evenly spaced ADI scores between 0 and 10 and, separately, for the median score in each of 10 equal-frequency deciles. For every point, we reported the posterior median with 50% credible interval (CI) and 95% CI. To ensure the robustness of our model choice, we conducted sensitivity analyses across 4 spatial-model specifications (Besag2 and BYM2 under both Poisson and negative‐binomial likelihoods). Bayesian inference was performed using integrated nested Laplace approximation (INLA) because it combines computational speed and accuracy for fitting latent Gaussian models. Analyses were conducted in R version 4.3.1 (R Foundation for Statistical Computing, Vienna, Austria) with the R-INLA package version 23.09.09. Detailed descriptions of model diagnostics, prior distributions, and sensitivity analyses are provided in the Supplementary Material 1.

### Ethics statement

The mortality data used in this study were provided by Japan’s Ministry of Health, Labour and Welfare and can be accessed for restricted research use upon request. Because the analysis relied exclusively on anonymized, aggregate data, no formal ethical review was required.

## RESULTS

**Figure 1.**
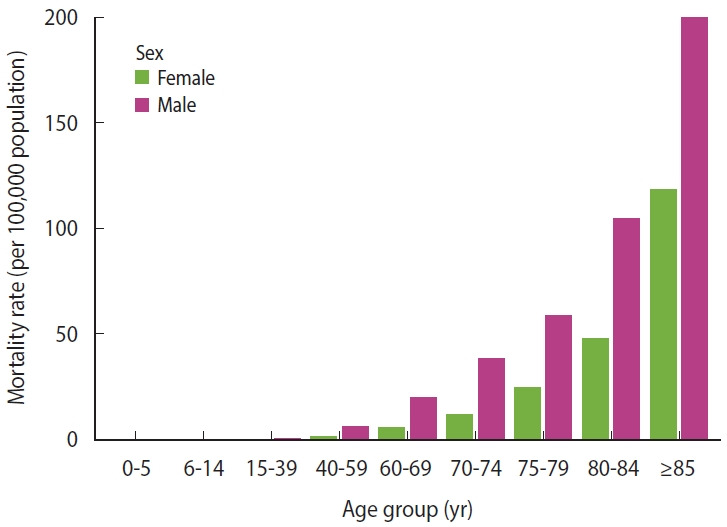
Age and sex-specific coronavirus disease 2019 (COVID-19) mortality rates in Japan per 100,000 population (2020-2021).

**Figure 2.**
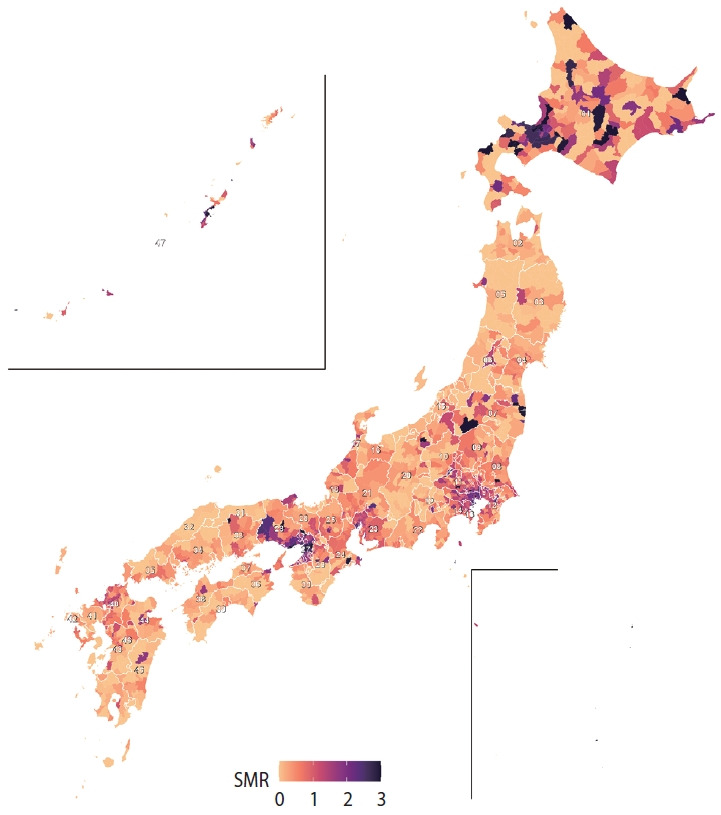
Map of standardized mortality ratios (SMRs) for coronavirus disease 2019 (COVID-19) across Japanese municipalities (2020-2021). The map reveals distinct geographic patterns in mortality risk, with elevated SMRs observed in northern prefectures including Hokkaido (01), Aomori (02), and Iwate (03), as well as central regions such as Niigata (15), Nagano (20), and Gifu (21). Western Japan also displays clusters of higher SMRs, particularly in Osaka (27), Hyogo (28), and Hiroshima (34). In contrast, lower SMRs are concentrated in southern regions, notably in Kyushu and Shikoku, and in central prefectures including Shizuoka (22) and Aichi (23). SMRs are displayed by quintile, with values greater than 3 truncated for visual clarity. Darker shades indicate higher SMRs, while lighter shades represent lower SMRs. The positions of Okinawa Prefecture and the Ogasawara Islands are geographically offset in the inset for improved visualization.

In 2020-2021, Japan recorded 20,760 deaths attributed to COVID-19, including 12,182 male deaths and 8,578 female deaths. Figure 1 presents the age distribution of these deaths by sex and shows that mortality rates increased with age, with male experiencing higher mortality than female in most age groups. Figure 2 maps SMRs across Japanese municipalities, colored by SMR quintile. Municipalities in Hokkaido, parts of the Tohoku region (such as Aomori and Iwate), and central areas including Niigata, Nagano, and Gifu showed elevated SMRs, indicating a higher RR of COVID-19 mortality. Western regions, including parts of Osaka, Hyogo, and Hiroshima prefectures, also displayed higher SMRs. Conversely, lower SMRs were predominantly observed in southern regions, including Kyushu and Shikoku, and in central areas such as Shizuoka and Aichi prefectures, as indicated by lighter shades. Global spatial autocorrelation was moderate in magnitude but highly significant (Moran’s *I*, 0.381; p<0.001), indicating that municipalities tended to resemble their neighbors in COVID-19 mortality risk. LISA refined this picture, showing that 9.8% of municipalities (n=186) belonged to significant clusters. Most were high-high “hotspots,” where a municipality with an elevated SMR was surrounded by similarly high-risk neighbors, underscoring shared local determinants of COVID-19 deaths. Hotspots were heavily concentrated in a handful of prefectures: Osaka (52 high-risk municipalities), Tokyo (29), Hokkaido (22), Hyōgo (19), and Okinawa (11), with smaller pockets in Saitama, Aichi, Kanagawa, Fukushima, Chiba, and Nara. In contrast, true low-low “cold-spots” were rare—only 2 municipalities in Nagano met this criterion.

### Spatial analysis of coronavirus disease 2019 mortality in Japanese municipalities

**Figure 3.**
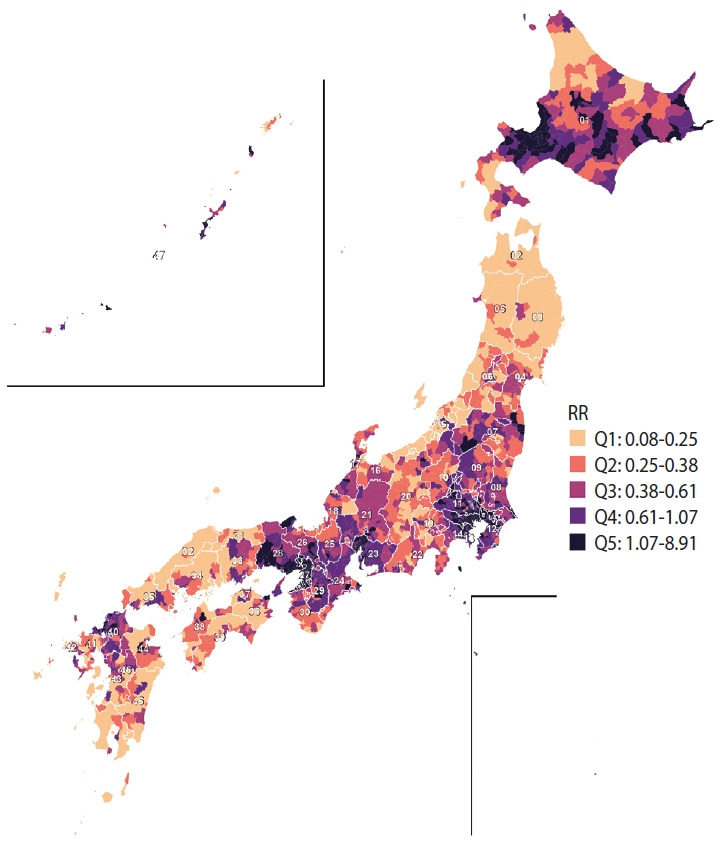
Estimated relative risk (RR) of coronavirus disease 2019 (COVID-19) mortality across Japanese municipalities (2020-2021). The map highlights geographic variations in RR, with elevated values observed in Hokkaido (01), particularly in its northern and eastern municipalities. High RR values also appear in parts of Kyushu and western Japan, including municipalities in Kagoshima (46), Miyazaki (45), Kumamoto (43), and Yamaguchi (35). While major urban centers such as Tokyo (13), Osaka (27), and Nagoya (23) generally exhibit moderate-to-low RR, some municipalities within these metropolitan areas show higher RR. Pockets of elevated RR are also observed in Okinawa (47) and remote island municipalities. RR values are displayed by quintile (Q), with the highest risks (Q5: RR >1.07) shown in dark purple to black and lower risks in lighter shades. The positions of Okinawa and remote islands are geographically adjusted in the inset for improved visualization.

Figure 3 shows the estimated RRs for COVID-19 mortality in each municipality, grouped into quintiles. Quintile 1 (0.08 to 0.25), shown in the lightest shade, indicates the lowest risk and is found mainly in scattered parts of central and southern Japan. Quintile 2 (0.25 to 0.38), in a slightly darker shade, appears throughout central Japan, with concentrations in the west and south. Quintile 3 (0.38 to 0.61), depicted in a medium shade, is more widespread nationwide, particularly clustering in central and western areas. Quintile 4 (0.61 to 1.07), represented by a darker shade, includes municipalities primarily in central Japan, with additional clusters in the north and west. The darkest shade, quintile 5 (1.07 to 8.93), denotes the highest risk and is prominently clustered in northern regions such as Hokkaido, as well as in several municipalities in central and western Japan. Overall, these patterns indicate substantial regional variation in the RR of COVID-19 mortality, with higher risks concentrated in northern, central, and western regions, and lower risks more common in southern and scattered central areas.

#### Fixed effects: ADI

**Figure 4.**
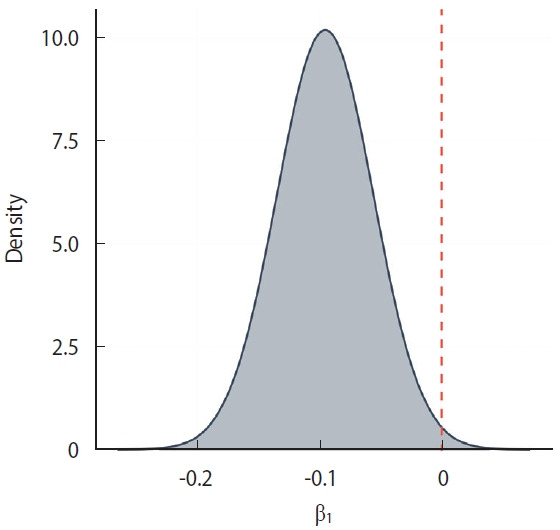
Posterior distribution of the Area Deprivation Index coefficient.

**Figure 5.**
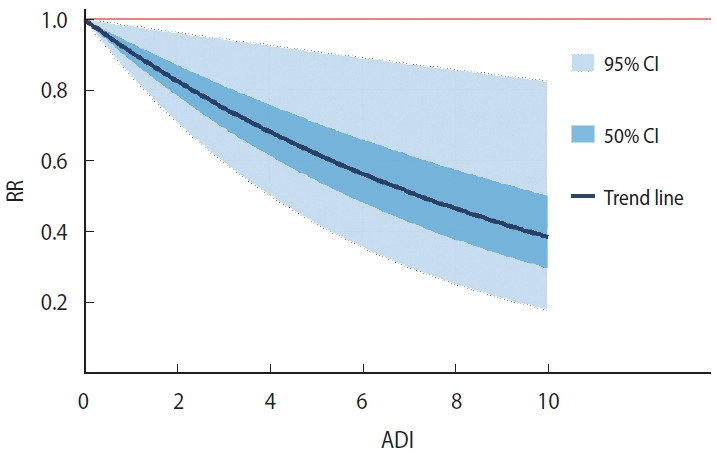
Relative risk (RR) of coronavirus disease 2019 (COVID-19) mortality across the full Area Deprivation Index (ADI) range in 1,894 Japanese municipalities, 2020-2021. The dark blue curve depicts the posterior median RR predicted by the Bayesian spatial model as ADI increases from 0 (least deprived) to 10 (most deprived). Medium blue shading shows the 50% credible interval (CI), while light blue shading indicates the 95% CI.

The regression coefficient for ADI was -0.095 (95% CI, -0.173 to -0.018), indicating that higher ADI values (greater deprivation) were associated with a lower log RR of COVID-19 mortality. This corresponds to an approximate 9.1% reduction in RR per 1-unit increase in ADI. The posterior distribution of the ADI coefficient is shown in Figure 4. To illustrate the association across the observed range of deprivation, Figure 5 presents the marginal effects, showing how RR varies with ADI. Sensitivity analyses indicated that alternative models (such as BYM2 and negative binomial) yielded similar ADI coefficients and CIs, whereas the selected Besag Poisson model provided the best fit (deviance information criterion, 7,692.93).

#### Random effects: spatial correlation

The model indicated substantial spatial correlation in COVID-19 mortality across municipalities, as reflected by the precision estimate for the spatially structured random effect (*τ_u_*=1.21). The precision of the unstructured random effect was estimated at *τ_v_*=3.74. The spatial variance ($\sigma_{u}^{2}$≈0.83) was substantially larger than the unstructured variance ($\sigma_{v}^{2}$≈0.27), implying that spatial effects accounted for approximately 75.5% of the total random variance.

## DISCUSSION

Our study revealed that an increase in the ADI is associated with a decrease in the RR of COVID-19 mortality within Japan. This suggests that more deprived areas had relatively lower COVID-19 mortality than less deprived areas, and our modeling indicates they were highly unlikely to have experienced greater mortality risk.

This finding contrasts with observations in other countries where area deprivation has been consistently linked to higher COVID-19 mortality. For instance, a similar analysis in Germany during 2020-2021, applying the same methodology, found higher COVID-19 SMRs in more deprived areas, especially during later infection waves [13]. In England, an analysis performed over a similar period showed that more deprived regions experienced higher mortality, highlighting “deprivation amplification” [14]. In the United States, after adjustment for patient-level characteristics (including comorbidities), the most disadvantaged neighborhoods had 74% higher odds of mortality, indicating a strong impact of socioeconomic factors. A global meta-analysis likewise found higher COVID-19 mortality in socioeconomically disadvantaged areas, reflecting the pandemic’s exacerbation of existing health inequalities [5]. Cross-national differences in the COVID-19 deprivation-mortality gradient appear to depend on how each country’s deprivation profile aligns with its pandemic governance structure rather than on cultural factors. In Japan, municipal public health centers (*hokenjo*) directed testing, tracing, and patient triage within a voluntary, request‑based legal framework [6,15]. The Japanese ADI mainly identifies sparsely populated rural municipalities, and these settings had lower transmission potential, consistent with the inverse gradient we observed [12]. England displays the opposite pattern: its Index of Multiple Deprivation captures urban crowding and public-facing occupations, and the most deprived quintile recorded about 50% higher COVID-19 mortality in the first 2 waves [14]. These risks were intensified by a decade of austerity that reduced local public health budgets most sharply in the poorest authorities [16]. In Germany, deprivation is also concentrated in dense urban districts, where COVID‑19 risk is higher [13]. Although the Robert Koch Institute set national guidance, implementation fell to the federal states (*Länder*) and often understaffed, under‑digitized local health offices (*Gesundheitsämter*), slowing containment [17,18]. Thus, Japan’s rural‑weighted deprivation profile plus agile local infrastructure blunted the deprivation effect on COVID‑19 mortality, whereas Germany’s urban‑weighted profile and fragmented local response amplified it.

Previous research by Yoshikawa & Kawachi [19] identified significant socioeconomic disparities in COVID-19 outcomes within Japan, with lower household incomes, higher unemployment rates, and other socioeconomic disadvantages linked to higher COVID-19 incidence and mortality. While their study provided valuable insights using prefecture-level data, substantial geographic variation exists within prefectures, and examining smaller geographic units may reveal additional patterns of socioeconomic impact. Our study used municipal-level data, enabling a more detailed analysis of deprivation and health outcomes. The contrasting findings between prefecture-level and municipal-level analyses highlight the importance of considering the ecological fallacy in spatial epidemiology. Prefecture-level data often mask important socioeconomic differences because of the scale at which variation occurs within prefectures [20,21]. Furthermore, the ADI used in our study, developed by Nakaya et al. [12], is a robust, validated measure of area deprivation and has been shown to correlate with all-cause mortality. The use of Bayesian spatial models, incorporating both spatially structured and unstructured random effects, strengthens our findings by accounting for geographic clustering and other spatial dependencies in the data.

The unexpected inverse relationship between the ADI and COVID-19 mortality in Japan can be attributed to several unique features of the Japanese healthcare and social support systems. These features ensured equitable access to both pharmaceutical treatment and non-pharmaceutical interventions (NPIs), which were critical in preventing deaths among patients with COVID-19 across all regions, regardless of socioeconomic status.

First, public health centers (*hokenjo* in Japanese), established by local governments, provide a wide range of services to local populations, including infectious disease care, nutrition support, and overall health maintenance. As of 2019, Japan had 472 such centers [22]. During the COVID-19 pandemic, *hokenjo* staff—particularly public health nurses—played an essential role in reducing mortality among the elderly population. These professionals were critical in identifying infected patients, ensuring their well-being, and promoting healthy behaviors during infection [6,23]. Reaching the elderly population, 20% of whom live in relative poverty, represents one vital task of these centers [24]. The proactive efforts of *hokenjo* staff in managing and supporting these vulnerable populations were instrumental in mitigating the pandemic’s impact, substantially reducing COVID-19 mortality among the elderly [23].

Furthermore, Japan’s legal framework was instrumental in its effective COVID-19 response. Early in the pandemic, COVID-19 was classified as a Class II infectious disease, providing a legal foundation for crisis management [25]. These provisions mandated that COVID-19 treatments, including hospitalizations and life-saving therapies, be publicly funded, eliminating out-of-pocket expenses and ensuring equal access to healthcare. Regional governance was vital to implementing this mandate. Japan’s health emergency governance system operates at 3 levels. The Ministry of Health, Labour and Welfare oversees the system nationally; prefectural health bureaus manage the regional level; and municipal health centers manage the local level. Under the Community Health Act, all cities and districts have established health centers responsible for local medical care, infectious disease prevention, and public health education [26]. During the COVID-19 pandemic, these centers implemented a cluster-based approach, conducting intensive investigations to identify and manage sources of superspreading events. This localized governance ensured that every region, including socioeconomically disadvantaged areas, had equitable access to necessary healthcare services [6,26].

While these institutional mechanisms effectively reduced disparities in healthcare access, they cannot fully explain our finding that areas with higher deprivation experienced lower mortality rates. We propose several structural and social characteristics of Japanese municipalities that may account for this paradoxical relationship. First, the geographic distribution of deprivation in Japan, as measured by the ADI, closely overlaps with rural areas, which have lower population density and correspondingly lower transmission risk [12]. These rural municipalities are characterized by limited public transportation networks and a high reliance on personal vehicles, naturally restricting cross-regional movement. This limitation became more pronounced during the COVID-19 pandemic, as elderly residents—who often rely on others for transportation—restricted their mobility in accordance with new social norms [27]. Second, the predominantly rural nature of these communities fostered stronger adherence to pandemic-related social norms, particularly among elderly residents, who showed high compliance with social distancing measures and gathering restrictions [27]. Finally, distinctive intergenerational living patterns in these regions likely served as a protective mechanism during the pandemic, as research has shown that rural elderly populations benefit from stronger social integration and more robust informal care networks than their urban counterparts, potentially facilitating better health monitoring and earlier medical intervention when needed [28]. These geographic, social, and structural characteristics—typically associated with deprivation in Japan—appear to have functioned as protective mechanisms against COVID-19 mortality when supported by robust public health infrastructure.

However, several important limitations must be acknowledged. First, the study’s ecological design confined us to area-level associations and precluded any causal inference at the individual or municipal level. A municipality that appears deprived yet records low mortality may still contain vulnerable subgroups whose risks are hidden by aggregation, and the inverse deprivation gradient observed should thus be interpreted as associative rather than causal. Country‑level studies in England and Germany demonstrate how strong individual‑level gradients can be masked or even inverted when spatial units change, reminding us that our inverse association cannot be interpreted as evidence that deprived individuals were protected. Furthermore, our design choices also limit generalizability. While focusing on 2020-2021 isolated the emergency‑declaration period, it also excluded the Omicron wave and the shift to “living with COVID-19,” when different mechanisms (such as large‑scale vaccination and behavioral fatigue) may have reshaped deprivation gradients. Likewise, we used the pre-pandemic ADI, which captures structural deprivation but not pandemic-specific exposures such as care-home density or essential-worker mobility. Accordingly, our findings best describe early-pandemic conditions and should be extrapolated to later periods with caution.

A critical methodological limitation is our inability to disaggregate mortality data by 5-year age groups. The relationship between age structure and area deprivation—particularly salient in Japan’s aging society—therefore remains only partially explored. Perhaps most importantly, we could not adjust for municipal-level vaccination rates, leaving a key potential mediator between area deprivation and mortality unexamined and preventing adjustment for socioeconomic differences in vaccine hesitancy [29]. Vaccine uptake and the intensity of local NPIs varied markedly across Japanese prefectures; omitting these pandemic-specific factors could bias the inverse deprivation gradient in either direction. Future work should therefore link fine-grained vaccination coverage with municipal or prefectural NPI stringency indices to disentangle these pathways [30,31]. Because the ADI is a composite of census variables, it cannot capture behavioral adaptations (e.g., mask use, mobility reductions) at the individual level; attributing our municipality-level effects to such behaviors thus risks cross-level bias. Finally, the fact that spatial random effects accounted for 76% of the residual variance signals the influence of substantial unmeasured regional confounders (e.g., healthcare capacity, dominant local industries, or transit patterns) that likely overshadowed the deprivation effect.

Our findings demonstrated that higher ADI levels were associated with lower COVID-19 mortality risk in Japan, contrary to global trends in which greater deprivation is usually linked to higher mortality. This inverse relationship, identified with a Bayesian spatial model applied to municipality-level data, highlights the effectiveness of Japan’s decentralized health system built around autonomous local public health centers. Japan’s legal framework ensured free COVID-19 treatment, and a strong culture of disaster preparedness fostered compliance with NPIs, promoting equitable access to care. Collectively, these features suggest that robust, universal access to healthcare can sever the expected link between deprivation and poor outcomes during a crisis. While these systemic factors helped reduce healthcare access disparities, the lower mortality in more deprived areas likely reflects additional protective factors—such as lower population density, different occupational structures, and community-specific behavioral adaptations—underscoring the need for individual-level analyses to disentangle the protective effects of rurality from those of specific public health interventions.

## Figures and Tables

**Figure f6-epih-47-e2025049:**
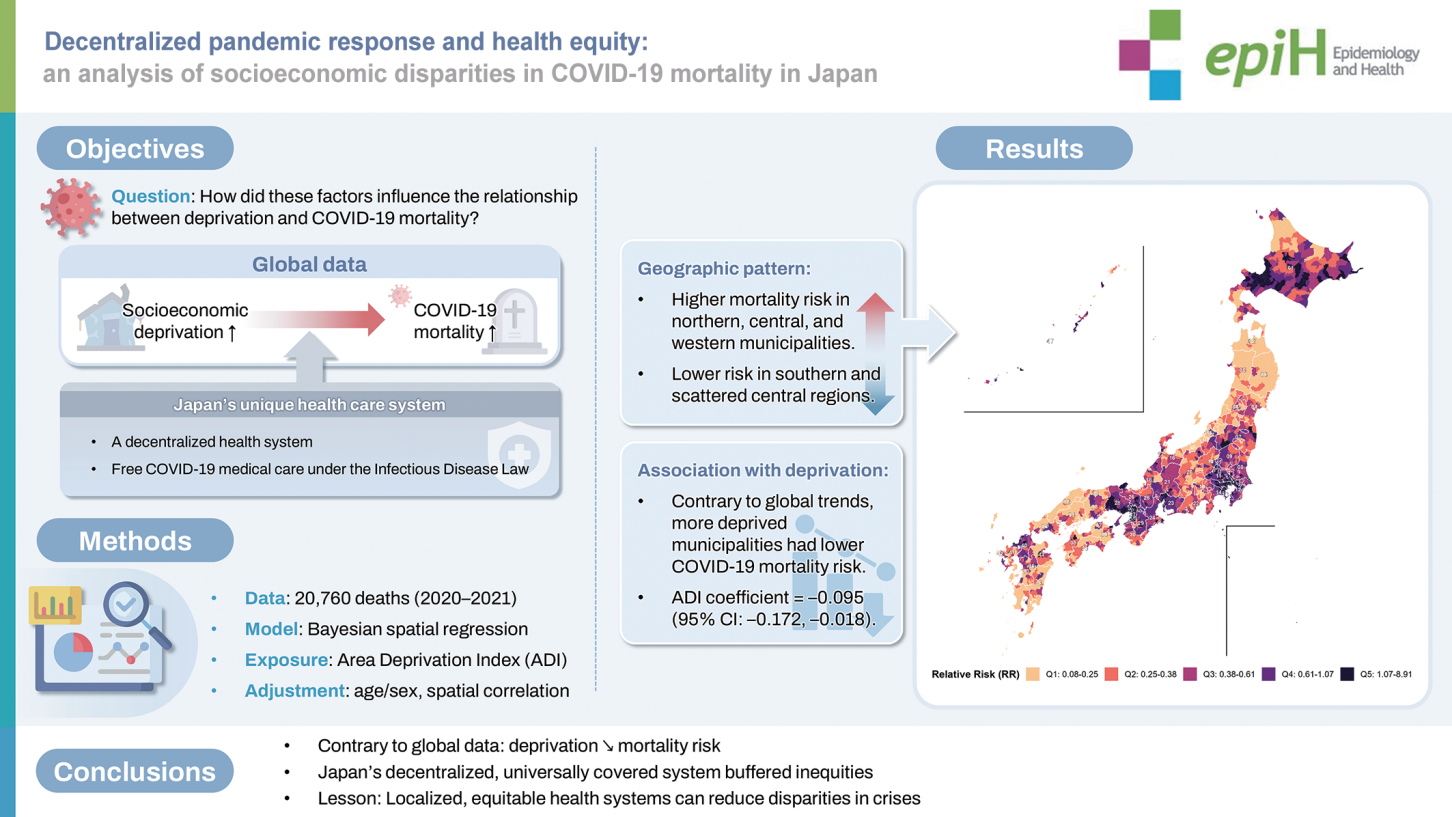


## Supplementary materials

Supplementary Material 1. Area Deprivation Index
