# Supplementary material for: Decentralized pandemic response and health equity: an analysis of socioeconomic disparities in COVID-19 mortality in Japan
Source: Epidemiol Health. 2025 Aug 28;47:e2025049. doi: 10.4178/epih.e2025049 (PMC12869140; doi:10.4178/epih.e2025049)
Supplement: Supplementary Material 1. — Area Deprivation Index [file epih-47-e2025049-Supplementary-1.docx]

**Supplementary Material 1.**

**Area Deprivation Index**

**Methods**

## Model Framework

Our primary objective was to model the observed number of COVID-19 deaths across different spatial units and to calculate the Relative Risk (RR). The model formulation incorporated Poisson regression, defined as:

$$Y_{m}\sim Poisson\left( E_{m},\theta_{m} \right)$$

where $Y_{m}$ represents the observed COVID-19 deaths in area m, $E_{m}$​is the expected number of COVID-19 deaths (used as an offset), and $\theta_{m}$​denotes the Relative Risk in area M.

$$loglog \left( \theta_{m} \right) =\beta_{0}+\beta_{1}\times{ADI}_{m}+u_{m}+v_{m}$$

$$for m=1,\ldots,number of municipalities included in the analysis$$

where $\beta_{0}$​ is the intercept (baseline risk), $\beta_{1}$​ is the coefficient for ADI, $u_{m}$ represents the spatially structured random effect, and $v_{m}$​ is the unstructured random effect.

### Spatial Structure and Random Effects

For the spatially structured random effects $u_{m}$, the Besag model was employed:

$$u_{m}\sim Normal\left( \sum_{j\in ne\left( m \right)} u_{j}/n_{m},\tau_{u}^{-1} \right)$$

Where $ne\left( m \right)$ indicates the neighboring areas of $m$, $n_{m}$ is the number of these neighbors. The precision parameter is denoted as $\tau_{u}$.

The unstructured random effects $\left( v_{m} \right)$ were assumed to be IID normal:

$$v_{m}\sim Normal\left( 0,\tau_{v}^{-1} \right)$$

Here, $\tau_{v}$ represent the precision of the unstructured component.

## Priors and Hyperpriors

Gaussian priors with a mean of zero and a large variance for the fixed effects, and log-gamma priors for the precision parameters of both the Besag model $\tau_{u}$ and the IID random effects ​$\tau_{v}$.

$$\tau\sim Gamma\left( 1, 0.00005 \right)$$

## Model Fitting

To conduct our Bayesian inference, we fitted spatial models using Integrated Nested Laplace Approximation (INLA). The fitting of the model was performed using the R- INLA package version 23.09.09 and R version 4.3.1.

# Model Diagnostics


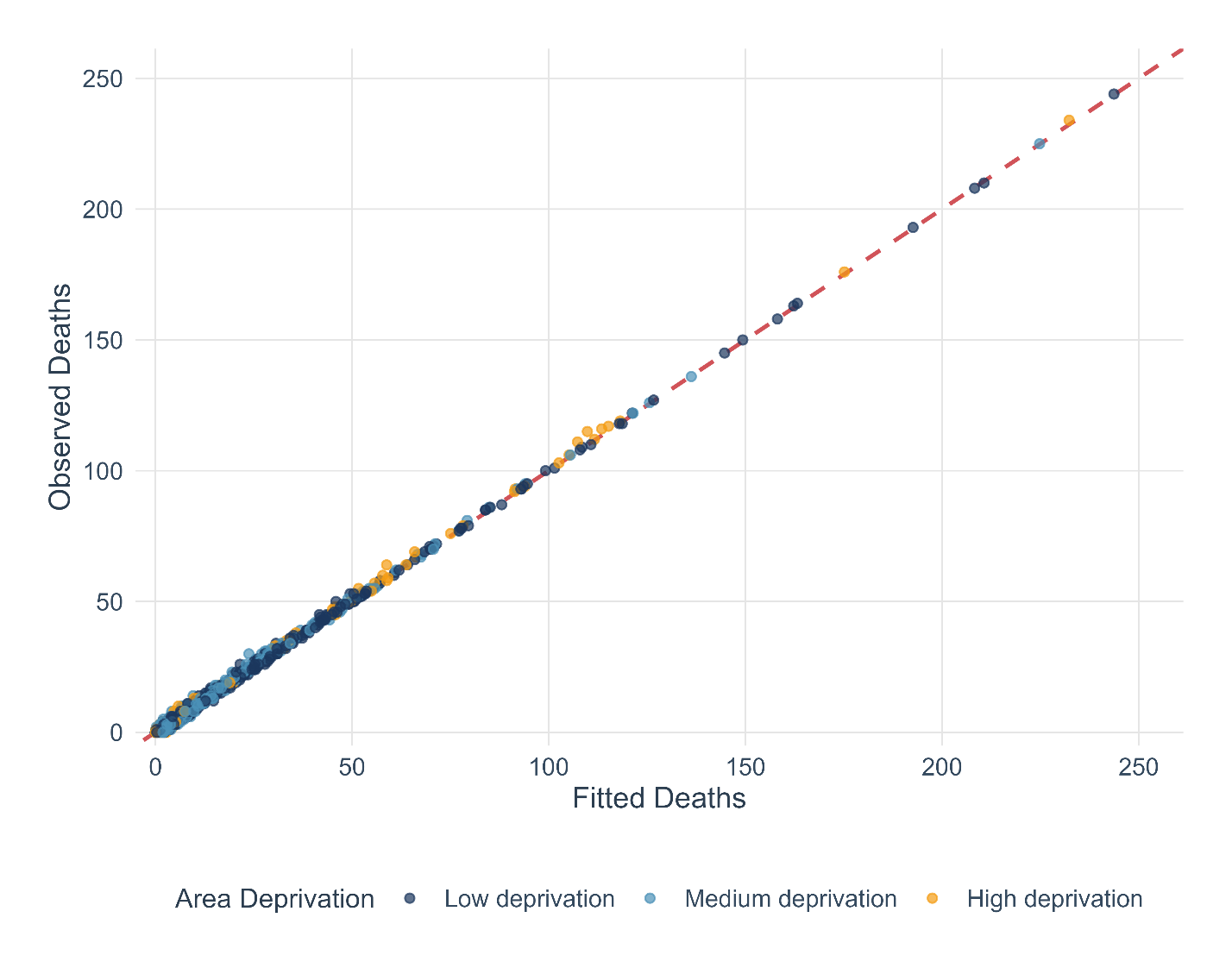


*Figure s1. Observed vs Model-Predicted COVID-19 Deaths by Municipality in Japan.*


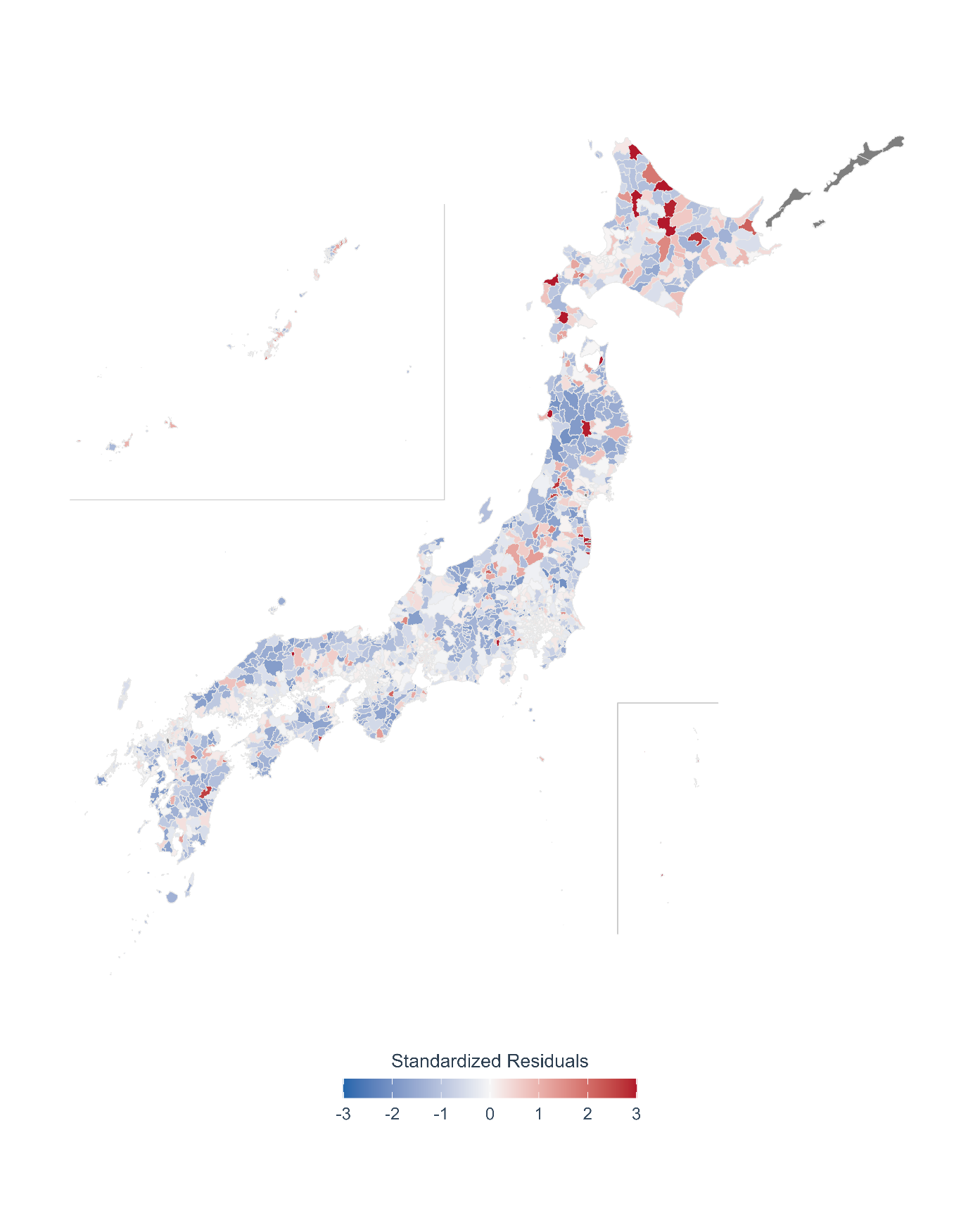


*Figure s2. Spatial distribution of standardized residuals from Bayesian spatial Poisson model of COVID-19 mortality across Japanese municipalities.*

# Supplementary Results


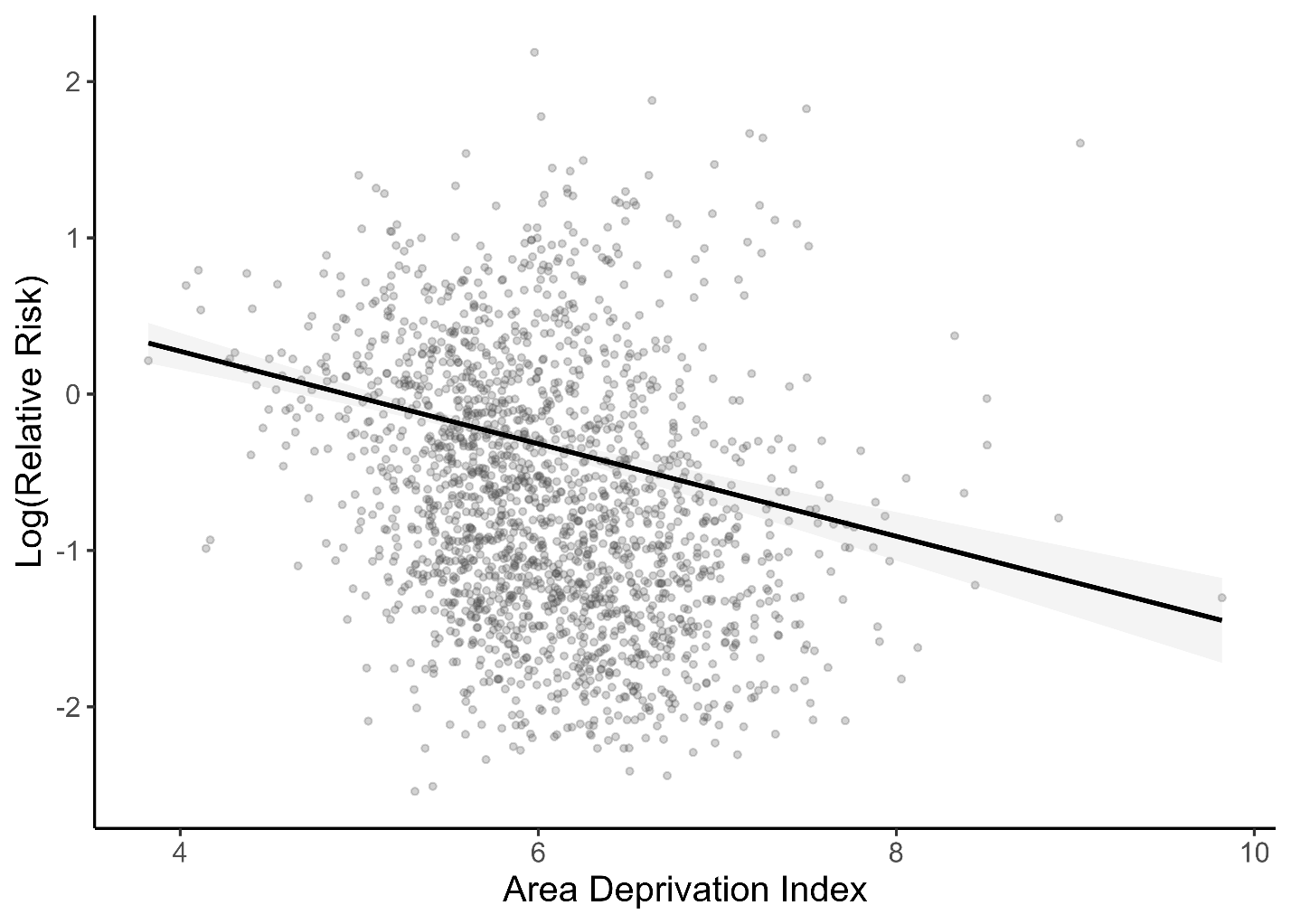


*Figure s3. Unadjusted scatterplot of observed log‑relative risk of COVID‑19 mortality versus Area Deprivation Index for all 1 894 Japanese municipalities, 2020–2021.*

## Sensitivity Analysis

*Table s1. Sensitivity analysis comparing different Bayesian spatial models using INLA framework*

| Model | Distribution | Model Fit (DIC)¹ | ADI Coefficient (β₁) | 95% Credible Interval |
| --- | --- | --- | --- | --- |
| Besag^2^ | Poisson | 7692.93 | -0.095 | (-0.173, -0.018) |
| BYM2 | Poisson | 7693.44 | -0.096 | (-0.173, -0.018) |
| Besag | Negative Binomial | 7925.45 | -0.095 | (-0.173, -0.018) |
| BYM2 | Negative Binomial | 7903.73 | -0.095 | (-0.173, -0.018) |
| *^1^* Lower DIC indicates better model fit  *^2^Besag model with Poisson Distribution was used in the research* | | | | |
